# Born or not: A moderated mediation model of the relationship between work-family conflict and female employees' wellbeing based on fertility intention during the COVID-19 pandemic

**DOI:** 10.3389/fpubh.2023.1093048

**Published:** 2023-02-28

**Authors:** Zhen Zeng, Jian Ma, Yueru Ma, Dayuan Li, Yong Sun

**Affiliations:** ^1^School of Business, Central South University, Changsha, Hunan, China; ^2^Xiangya Hospital, Central South University, Changsha, Hunan, China

**Keywords:** work-family conflict, wellbeing, fertility intention, husband's share of housework, husband's flexible work pressure

## Abstract

The previous academic research on work-family conflict mainly focused on the relevant elements in the work field. This study concludes that elements of the family domain have a significant impact on the relationship between work-family conflict and employee wellbeing. Female employees' perceptions of wellbeing largely depend on their willingness to have children when they take on family roles. During COVID-19, employees had more time to fulfill both work and family roles in the family sphere due to the epidemic blockade, the contribution of the female employee's significant other (husband) in family matters had a significant impact on Fertility intention. This study using SPSS 24.0 AMOS 20.0 and M plus 7.4 statistical analysis tools to test the proposed hypotheses. In the paired data of 412 working female employees and husbands of Chinese dual-earner families with different occupational backgrounds, hypothesis testing results support that female employees' work → family conflict is negatively related to female employees' fertility intentions, and female employees' fertility intentions are positively related to wellbeing; female employees' family → work conflict is negatively related to female employees' wellbeing; husband's flexible work stress is negatively related to husband's share of housework; husband's share of housework moderated the front, rear and overall mediating effects by the fertility intention. When formulating policies, the managers should consider not only the direct effects of policies, but also the indirect effects that policies may have on other family members of employees. Managers should develop management policies during an epidemic that are more responsive to the actual needs of employees during an epidemic. The management of female employees should give due consideration to the family status of female employees and the enterprises should recognize the importance of childcare for female employees.

## 1. Introduction

Employees are an important part of the organization. Although both work and family aspects are of great importance to employees, research on work aspects has always occupied an important place in the study of organizational behavior, while research on employees' family aspects has lacked attention. However, the goal of organizations is not only to focus on performance improvement, but also on the wellbeing of individual employees and their families, so certain characteristic factors at the employee family level, such as the impact of fertility intentions on the relationship between work-family and wellbeing, have unique research value.

In the past 30 years, China has maintained rapid economic growth under the socialist system, which is mostly based on China's demographic dividend. However, according to the data released by the National Bureau of Statistics of China, the birth rate of China in 2020 was 8.52 per thousand, which is lower than 1%, a new low in more than 40 years. The number of people born in 2021 was dropped by 1.38 million compared with 2020. The decline of the fertility rate will first lead to the slowdown of economic development and the transformation of economic growth (from labor-intensive production to technology intensive production). At the same time, this study believes that the decline in fertility will also lead to changes in family wellbeing, more specifically, it will affect the wellbeing of women in the family, because women have traditionally assumed more family responsibilities than men. Meanwhile, China's fertility policy has undergone many major changes. The attitude and understanding of Chinese female employees toward fertility are also changing significantly with social development and economic growth.

China has adopted epidemic control measures different from those of other western countries. From the outbreak of COVID-19 epidemic in December 2019 to December 2022, the Chinese government has always adopted the policy of combining nucleic acid detection with double code identification of health code and journey code, and implementing regional blockade and dynamic zero clearing of “confirmed patients” in epidemic areas (communities). This series of control measures made the flexible work frequency of Chinese employees in home isolation much higher than that of employees from other countries in the same period. This is why this study should consider the impact of the husband's flexible work pressure on the husband's housework in the context of the epidemic. Especially after the outbreak of the COVID-19 epidemic, China is one of the few countries in the world that took the lead in recovering from the epidemic. As a special sample of the global economy, China has attracted many scholars' attention and research, especially in the field of human resources and organizational behavior. In recent years, many scholars have paid attention to how the Chinese government and enterprises can effectively organize and manage human resources under the major global public health crisis, so as to achieve efficient recovery of economic production.

The COVID-19 epidemic has caused heavy damage to the global economy and people's production and life. Against this historic background, all countries are trying to do their utmost to control the epidemic and let the country and people get rid of the negative impact of the COVID-19 epidemic as soon as possible. From a macro perspective, investment, consumption and export, the troika driving China's economic growth, have weakened during the epidemic. From the government's standpoint, whether it is to stimulate investment, promote consumption or expand domestic demand, it is inseparable from human resources to provide production, construction and product services. People are an important factor of production. Under the background of the normalization of the epidemic situation, the government and enterprises are eager for employees to participate in social labor and drive economic recovery and enterprise revenue growth. From the micro level, first of all, the large-scale shutdown caused by COVID-19 has led to the unemployment of most employees in a country without a source of income, which has seriously affected the quality of life of individual employees (1). During the epidemic, employees will be unemployed, but their housing and car loans for living still need to be repaid continuously. Life is still going on, and all daily expenses need to be sustained by continuous income, even when employees are isolated at home. Secondly, the social isolation during the prevention and control of COVID-19 epidemic led to the lack of basic offline social activities and communication among employees, which caused mental stress on individual employees and affected their individual health to a certain extent (2). Some data show that at the beginning of the liberalization of the isolation measures in different cities in China, people will generally have a wave of retaliatory consumption (3). Some studies have pointed out that retaliatory consumption is a release of mental pressure (4). Based on the above, individual employees in the context of COVID-19 epidemic have a strong desire to return to social production and commercial activities. Therefore, work is particularly important to employees, both male and female. In modern society, men, as the main source of family income, dominate the family economy. At the same time, women have contributed more value to all aspects of social and economic activities—production, service, circulation and other fields than in any previous human historical period. But at the same time, women's responsibilities in the family have not changed at all. They give birth to children, raise children, and become the most important partner and learning mentor of children. Nowadays, elite education is prevailing in China, The demands of female employees on childcare in their families—whether from the social competitive environment or from the improvement of their own education level—are increasing. According to the Global Gender Gap Report 2020 (5) released by the World Economic Forum, in 2019, China's female education score has risen to 0.973 points, ranking first in the world with an indicator score close to 1.00 which is full equality. Based on the principle of limited individual resources of the conservation of resource theory, the multi role pressures and conflicts encountered by female employees in the new era from job requirements, childcare requirements, household requirements and other requirements will inevitably lead to further intensification of the scarcity of female employees' individual resources, which will cause changes in female employees' expectations for fertility. From the 1970s and 1980s when Chinese wanted to be born but policy restrictions could not, to the present when people do not want to be born but the national policy has been born, China's population policy has undergone a major change. Specifically, China implemented a comprehensive two child policy in 2016, completely abolishing the family planning policy implemented since 1978. China's population issue has always been a global, long-term and strategic issue. In 2021, the report on the work of the Chinese government at the two sessions of the National Congress and the Chinese People's Congress again emphasized optimizing the birth policy to cope with the aging of the population. At the meeting of the Political Bureau of the CPC Central Committee, it was decided to implement the three child birth policy. This series of population policy adjustments is a concrete manifestation of the self-evolution and evolution of China's human resources in terms of quantity and quality brought about by China's economic growth and rapid social development.

China's population is about to enter the era of negative growth, and the continuous decline of the birth rate will further restrict China's economic growth from the supply side and the demand side (6). On the one hand, the shift of economic growth momentum to innovation driven may aggravate the asymmetry between employment creation and employment destruction, the imbalance between human capital supply and demand, and increase the natural unemployment rate dominated by structural and frictional factors; on the other hand, insufficient employment and low quality are not conducive to the reasonable improvement of workers' remuneration, and will also hinder the full play of China's economic growth potential from the demand side.

Based on the conservation of resource theory, this study constructs a two-stage mediated model with the husband's share of housework as the moderating variable, fertility intention as the mediating variable, and examines the relationship between work-family conflict and their wellbeing encountered by female employees in Chinese society. The mechanism and boundary conditions of its generation, taking COVID-19, China's two-child and three-child fertility policy as the background. A questionnaire was used to collect paired data from 412 working female employees with different occupational backgrounds in Chinese dual-earner families and their husbands, and data analysis was conducted using SPSS 24.0 AMOS 20.0 and Mplus 7.4 statistical analysis tools to test the proposed hypotheses.

The findings not only deepen the understanding of work-family conflict and wellbeing research, but also clarify the mechanism of work-family conflict on wellbeing at the family level, expand the research field of work-family conflict, highlight the important influence of work-family conflict on wellbeing in the family context, and provide theoretical guidance and practical insights for managers to comprehensively understand the influence of work-family conflict on employees.

## 2. Review of relevant studies

Role conflict may occur when working employees occupy multiple roles in the work and family domains and are unable to handle demanding work and family roles at the same time. Work-family conflict is defined as “an inter-role conflict in which role pressures from the work and family domains are incompatible in some way” (7). Work-family conflict is a two-way street; work → family conflict refers to work-role interference with the family role, while family → work conflict refers to family-role interference with the work role (8). The concept of work-family conflict provides a useful theoretical perspective on the declining fertility of employed women. Previous research has shown that female employees experience more work → family conflict than male employees due to unequal division of labor in the family, which is considered to be the main influencing factor for the low fertility rate of female employees (9). That is, to increase female employees' fertility intentions, work-family conflicts caused by gender inequality in the family domain should be addressed (10). Becoming a parent is both rewarding and hard, and both parents feel happy and take responsibility for raising their children (11). Parenting requires sustained energy, and the time and energy taken up requires working parents to correct their daily behaviors or perceptions (12). Working parents tend to experience particularly high levels of parenting stress as the burden of childcare increases and as roles conflict between the work and family spheres (13). Abidin's (14) model of parenting stress suggests that barriers to parent-child interaction, perceived difficulties in child communication, and parents' expressed complaints about life in their daily lives can negatively impact parenting. As with work-family conflict, parenting stress is considered an indicator of reduced fertility intentions among working female employees (15). Parents with jobs are more likely to be discouraged from having a second child if parenting stress continues at a high level and is accompanied by negative emotions such as depression and anxiety (16). Considering that high levels of parenting stress among working mothers who already have one child may reduce their willingness to have a second child, understanding how to reduce parenting stress among working women employees is an important issue in low-fertility countries (17). Work → family conflict and childcare stress are considered to be the main reasons for low fertility among working female employees in European countries (13), but few studies have examined these associations in Chinese samples. In addition, mothers' parenting experiences at the time of their first child, including the amount of “parenting support” they received from their husbands, and their work status upon returning to the workplace (work-family conflict) are strongly associated with their second childbearing intentions. Given that parenting support from working fathers helps to alleviate work-family conflict and parenting stress among working mothers (18), if working fathers can spend more time and energy at home and support working mothers (female employees) by engaging in family activities such as housework, this may reduce work-family conflict and parenting stress among working mothers This in turn increases their motivation to have a second child.

In previous studies, control variables such as socioeconomic status (e.g., age, educational background, income) of working female employees were found to be strongly associated with their intention to have children. Younger female employees have higher intentions to have children than older female employees, and these results may be related to biological reproductive capacity (19). Increased education of female employees also somewhat reduces female employees' intention to have children, due to the fact that highly educated female employees are more likely to be economically active and have a strong desire for self-fulfillment (20). Other studies take the opposite view, arguing that more educated female employees tend to have more children than less educated female employees because educated women are likely to marry men of higher socioeconomic status (21). In addition, researchers found that female employees' income was positively associated with fertility intentions (22).

During the continuous crisis pandemic of COVID-19 period, policy makers and organizations should focus on formulating and implementing policies to promote family friendly workplaces, so as to enhance employees' sense of corporate belonging (23). The implementation of flexible work arrangements (FWA) provides opportunities for organizations to reduce staff mobility and promote employee development through work life balance plans (24). During the epidemic, the organization continuously strengthened its online psychological support for employees through the use of communication technology, which was also achieved through smart phone applications that could provide effective intervention functions (25). In addition, the results of a study taking German families as a sample show that various control measures against COVID-19 pose a more common threat to the quality of relationships between couples within the family and the healthy family functions (26). Some scholars believe that the work life conflict of employees has decreased in general during the epidemic, but these changes are also limited by the age of the youngest child in the family and the degree of work family integration (27). At present, the research on the work-family relationship of employees has not yet involved how the fertility intention is affected by family support related factors in the context of COVID-19. Based on the COVID-19 background and China's unique fertility policy changes, how does fertility intention affect the relationship between work-family conflict and female employees' wellbeing. In conclusion, based on the background of the COVID-19 and China's unique fertility policy changes, this paper intends to study how fertility desire affects work family conflict relations and the well-being of female employees, to supplement the research gaps in this area.

Based on the above, this study developed a scale to investigate the fertility intentions of female employees in the context of the “comprehensive two-child” and “three-child” policies and in the epidemic environment, based on the quantitative study of fertility intentions conducted by Miller (28) and Zheng (29). Based on the work stress scale compiled by Price and Spence (30), we developed a stress scale for flexible work situations during the epidemic to investigate the stress of flexible work (WFH) for husbands during the epidemic, including work-related stress and work-related stress (e.g., the need to punch in daily health codes and trip codes, and to complete nucleic acid tests on time). Five hundred dual-earner couples were invited to participate in the study, with husbands completing the husband's flexible work stress Scale and questions on the husband's time spent on housework; wives mainly completed the questionnaire's answers to the scale items on work-family conflict, family-work conflict, fertility intention, and wellbeing of female employees. The willingness of one of the spouses to participate was mainly solicited through an online WeChat group, and then the scales were sent separately after obtaining the consent of both parties through internal coordination between the spouses. The screening of a couple is identified by one spouse identifying the other's WeChat name.

## 3. Theoretical basis and research hypothesis

### 3.1. Work-family conflict and fertility intentions

**Figure 1:**
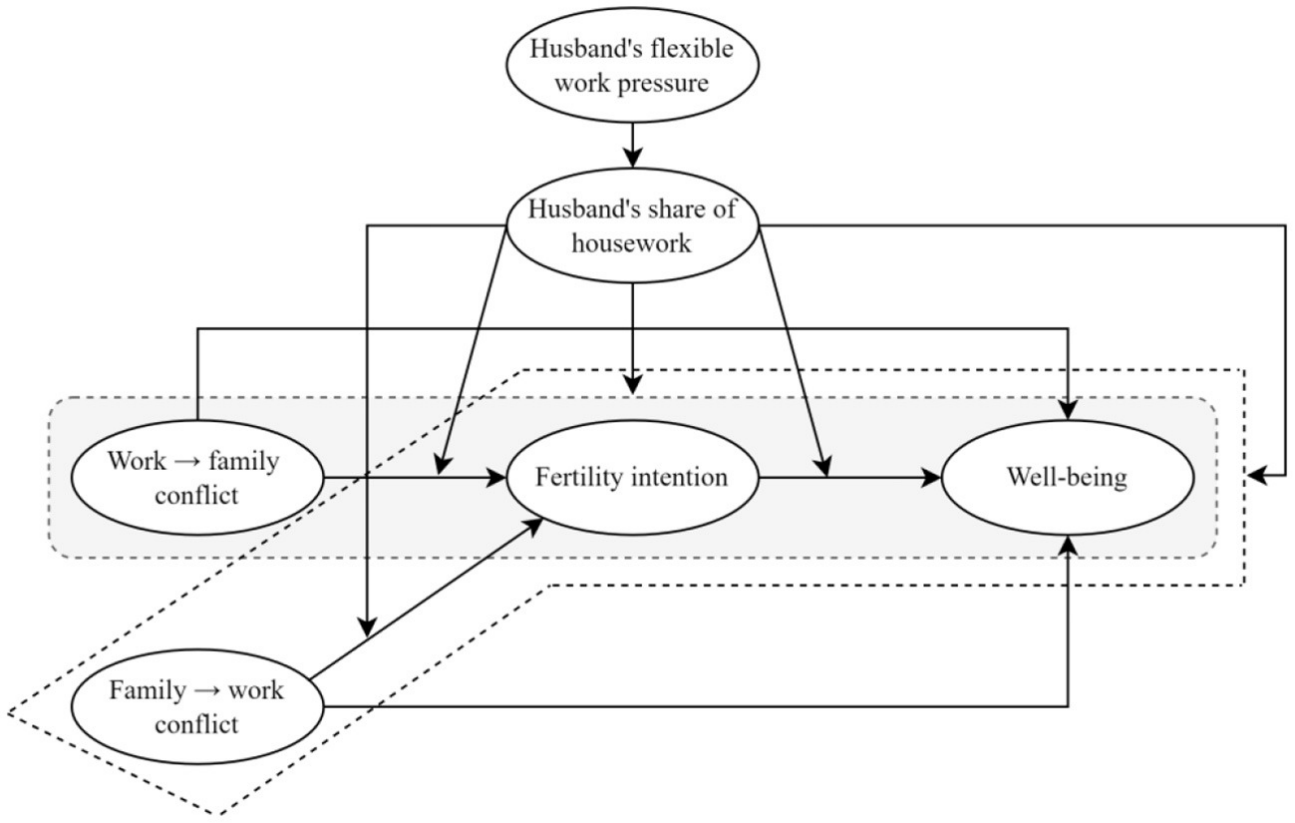
Hypothetical model.

Role conflict arises when individuals are unable to balance the rationing of resources between different roles. Unlike role conflict within the work domain (e.g., an employee acting as a leader or organizer of one project while acting as a performer of another), work-family conflict is a conflict that results when an employee is unable to reconcile the allocation of energy, time, and behavior between work and family, and is unable to achieve a balance between these two compartmentalized domains. Conservation of resource theory (COR-Conservation of Resources) (31) proposes that individuals are motivated to acquire, maintain, and protect resources, such as self-esteem, socioeconomic status, and employment, in order to manage work and family demands. Based on resource preservation theory, female employees who experience work-to-family conflict (being too busy at work to go home rarely to care for their sick father) or experience family-to-work conflict (being unable to accept a promotion offered by their supervisor because of the amount of time and energy required to care for their young children, because the promotion means more work tasks and more work pressure), their efforts to protect available resources (such as job opportunities or family relationships) will reduce the behaviors associated with continuing to generate this conflict, along with lower behavioral motivation, such as willingness to have children. Ma and Jin (32) concluded that work-family conflict has a significant effect on nurses' willingness to have a second child during their reproductive years. In summary, according to conservation of resource theory, childbirth threatens or consumes individual employees' time, energy, and personal resources, and due to the limited resources, female employees, based on their existing work-family conflict, will reduce their “willingness to have children” that may lead to increased work-family conflict in order to mitigate that conflict and protect the work-family relationship. Therefore, this study proposes hypotheses for a broad group of female employees in a wide range of occupational types based on their willingness to have children regardless of whether they have one, two, or three children. Therefore, the following hypotheses are proposed based on the female employees' fertility intention as shown in Figure 1.

**Hypothesis 1**. Work-family conflict among female employees is negatively associated with fertility intention.
**Hypothesis 1a**. Female employees' work → family conflict is negatively associated with fertility intention.
**Hypothesis 1b**. Female employees' family → work conflict is negatively associated with fertility intention.

### 3.2. Fertility intention and wellbeing

Fertility intention is the manifestation of fertility desire of individuals or families taking into account constraints, including specific dimensions such as the desired number of children, gender, and timing of childbirth (33). Fertility intention is a necessary but not sufficient condition for generating fertility behavior, and there is a definite link between the two. This study focuses on the expected number of children to examine fertility intentions and its impact on female employees' wellbeing.

Diener and Emmons (34) argues that “wellbeing” or “subjective wellbeing” refers to “people's evaluation of their lives,” including “cognitive judgments of satisfaction” and “affective evaluations of emotions and moods.” Cognitive judgments of satisfaction” and “affective evaluations of emotions and moods.” Boehm and Lyubomirsky (35) considers “happy people” to be “people who regularly experience positive emotions,” such as “wellbeing” such as “joy,” “wellbeing,” and “contentment.” Although wellbeing is important for both individuals and organizations (36), there is limited research on employee wellbeing (37). In this paper, people build on previous work, this study defines female employees' happiness as a comprehensive feeling of subjectively perceived wellbeing of female employees by integrating various experiences in the personal, family and work domains. Although the work role is one of the important social roles of female employees, their non-work roles (wife and mother) are also an important part of their lives. Excellent family roles also bring happiness to female employees.

It has been suggested that good family relationships have an important impact on individuals' perceived wellbeing (38). In family relationships, childbearing and the number of children are important aspects in reconciling family relationships (39). Studies point out that people with children are generally happier than those without children (40); having two or more children is happier than having only one child (41). In addition, the higher the number of children, the higher the individual's life satisfaction (42), and the number of children is positively related to the mother's wellbeing (43). At the same time, the wellbeing of the elderly is in an inverted U-shaped relationship with the number of children (44). Based on the logic that fertility outcomes are generated by fertility intentions, and based on the theoretical and empirical foundations of the above literature, this study proposes the hypothesis that. Based on the theoretical and empirical basis mentioned above, the following hypotheses are proposed as shown in Figure 1.

**Hypothesis 2**. Fertility intention is positively related to female employees' wellbeing.

### 3.3. Work-family conflict and wellbeing

For employees, life and work are two inseparable and important aspects. Since employees have limited energy and time, their energy and time consumption in one situation (e.g., work situation) will crowd out and spill over to the energy and time consumption needed in another situation (e.g., family situation). Based on the segmentation theory (45), it is commonly believed that work and family are conflicting fragmented in individuals. Work-family conflict is divided into two directions: “work → family conflict” and “family → work conflict” (46). The “work-family conflict” refers to the work stress, time and energy occupation generated by the work field, which adversely affects the family field. For example, a male employee has no time to take care of his wife during her illness and hospitalization due to high work pressure and heavy workload, so much so that it affects his family life, such as leading to the weakening of the couple's relationship and deterioration of the couple's relationship. “Family → work conflict” refers to the negative impact on employees' performance in the workplace due to the time and energy they need to pay for their family responsibilities. For example, a female employee cannot accept a business trip because she has to take care of her mother who is paralyzed at home all year round. Wellbeing is a measure of employees' survival status, and in the past literature its mostly related to job satisfaction and family satisfaction. With the development of economy and social progress, organizations are paying more and more attention to improving employee wellbeing, i.e., wellbeing, especially high-tech companies are doing a better job of humanistic care for their employees (47). Companies expect to attract and retain talents by improving employees' wellbeing and forming “word of mouth” and talent gathering effect in the industry (48). Regarding the relationship between work-family conflict and wellbeing, some scholars point out that work → family conflict is significantly negatively related to wellbeing, and family → work conflict is also significantly negatively related to wellbeing (49). Based on the above, the following hypotheses are proposed as shown in Figure 1.

**Hypothesis 3**. Work-family conflict of female employees is negatively related to the wellbeing of female employees.
**Hypothesis 3a**. Female employees' work → family conflict is negatively related to female employees' wellbeing.
**Hypothesis 3b**. Female employees' family → work conflict is negatively related to female employees' wellbeing.

### 3.4. Mediating role of fertility intentions

In conservation of resource theory, individuals lose resources in one domain, and out of the initial desire to protect resources and prevent their continued depletion, individuals take steps to reduce their continued investment of resources in that domain and instead invest them in other alternative domains that may yield resource gains (50). When female employees navigate between work and family and find that the behaviors, time, and stress generated by the family domain create significant conflicts in the work domain, female employees may reduce their resource investment in the family domain to reduce the conflicts, thus achieving the goal of reducing further resource depletion. In China, female employees' childbirth is one of their important matters in the family domain (51). Reducing the resource investment in the family domain means that female employees may be less willing to have children and reduce their reproductive behavior. And based on traditional Chinese values, the main social duty fulfillment and social value of women is to pass on the family line and bear children. The decrease in resource input at the family interface resulting from work-family conflict and the resulting decrease in fertility intention will prevent female employees from gaining recognition of traditional social universal values and reduce self-worth recognition, which will ultimately have a negative impact on the subjective wellbeing they may experience. Based on the above, the following hypotheses are proposed as shown in Figure 1.

**Hypothesis 4**. Female employees' fertility intentions mediate the relationship between female employees' work-family conflict and female employees' wellbeing.
**Hypothesis 4a**. Female employees' fertility intention mediates the relationship between female employees' work → family conflict and female employees' wellbeing.
**Hypothesis 4b**. The mediating role of female employees' fertility intention between female employees' family → work conflict and female employees' wellbeing.

### 3.5. Husband's flexible work pressure and husband's share of housework

Segmentation theory suggests that work and family are two separate domains, so the more time and energy employees spend and take up in the work domain, the less time and energy they must allocate to the family domain (45). During the epidemic, many husbands were required to work from home or work in isolation in hotels due to the local epidemic control measures because they were in a high outbreak area or their health code was red or yellow. Flexible work is a system in which employees can flexibly and autonomously choose their own work schedules in lieu of uniform, fixed commuting hours, provided that they complete their required tasks or work a fixed length of time (52). Regarding the work stress in flexible work status, Yue (53) believes that flexible work can reduce commuting stress for employees. At the same time, since supervisors cannot supervise employees' work completion progress on site, in order to avoid situations such as slacking off, supervisors may ask employees to complete their work tasks within more demanding working hours during flexible work, which may make employees' perception of stress stronger. When a husband experiences flexible work treatment during an epidemic, he is likely to redistribute his work and household time based on different perceptions of stress based on segmentation theory. Based on the above, the following hypotheses are proposed as shown in Figure 1.

**Hypothesis 5**. Husband's flexible work stress is negatively associated with the husband's share of housework, i.e., the higher the husband's flexible work stress, the lower the husband's share of housework.

### 3.6. Moderating effect of the husband's share of housework

It has been shown that context has a significant moderating effect (54). The family environment is one of the contexts that female employees encounter at home. When the home environment, presents a good family atmosphere, female employees can feel supported by the family. Talukder (55) found in his study that the work-family climate effectively moderates the relationship between family-supportive supervisory behavior and work-family balance. Family support includes family behaviors made by all other family members that can reduce the stress, time loss, and energy loss experienced by female employees in the family interface. For example, grandparents helping with child care and husbands having more time at home to help with child care or housework are considered typical family supports. Grandparent child care can shorten the birth interval between second children for women of childbearing age (53). In the context of the three-child policy, family support can alleviate work-family conflicts between the sexes (47). Husband's responsibility for housework is a typical form of family support, and since the literature supports that “context has a significant moderating effect,” the following hypothesis is proposed. Based on the above, the following hypotheses are proposed as shown in Figure 1.

**Hypothesis 6**. The relationship between female employees' work-family conflict and female employees' fertility intentions is negatively moderated by the husband's share of housework, i.e., the higher the degree of husband's share of housework, the weaker the effect of female employees' work-family conflict on female employees' fertility intentions.
**Hypothesis 6a**. The relationship between the husband's share of housework negatively moderates the relationship between female employees' work → family conflict and female employees' fertility intentions, i.e., the higher the degree of husband's share of housework, the weaker the effect of female employees' work → family conflict on female employees' fertility intentions.
**Hypothesis 6b**. The relationship between female employees' family → work conflict and female employees' fertility intentions is negatively moderated by the husband's share of housework, i.e., the higher the degree of husband's share of housework, the weaker the effect of female employees' family → work conflict on female employees' fertility intentions.

Based on the conservation of resource theory, individuals always try to protect and maintain existing resources and continuously acquire more new resources based on existing resources to reduce the net loss of resources (50). Since individuals will acquire resources through different channels and different resources affect individuals simultaneously, the magnitude of the effect of female employees' work-family on female employees' wellbeing depends on whether the resources lost in the process of generating the desire to have children and the resources acquired through other channels can be balanced when they offset each other. When husbands take up a certain proportion of housework, the loss of resources such as energy and time sacrifice that female employees should traditionally make in the family is compensated by their husbands' behavior. Female employees feel stronger resource support psychologically and get more time and energy resources in reality. Therefore, the greater mental pressure carried by female employees due to the desire to have children will be reduced, and the time and energy loss (resource loss) in the work interface and family interface due to the desire to have children will be reduced, and the resulting resource balance will strengthen the wellbeing of female employees to some extent. Based on the above, the following hypotheses are proposed as shown in Figure 1.

**Hypothesis 7**. Husband's share of housework negatively moderates the indirect effect of female employees' work-family conflict on female employees' wellbeing through fertility intention, i.e., the higher the degree of husband's share of housework, the weaker the mediating effect of fertility intention on work-family conflict and female employees' wellbeing.
**Hypothesis 7a**. Husband's share of housework negatively moderates the indirect effect of female employees' work → family conflict on female employees' wellbeing through female employees' fertility intentions, i.e., the higher the degree of husband's share of housework, the weaker the mediating effect of female employees' fertility intentions on work → family conflict and female employees' wellbeing.
**Hypothesis 7b**. Husband's share of housework negatively moderates the indirect effect of female employees' family → work conflict on female employees' wellbeing through female employees' fertility intentions, i.e., the higher the degree of husband's share of housework, the weaker the mediating effect of female employees' fertility intentions on family → work conflict and female employees' wellbeing.

Based on the conservation of resource theory, individuals must invest more resources in the matters they undertake in order to prevent further depletion of resources to compensate for the loss of resources (50). Female employees may experience greater time and energy depletion in the process of generating fertility intentions due to concerns about the birth of future children, thus generating more stress. However, different family contexts give different resource allocations to female employees, and when the family context is in a supportive state, i.e., family support behaviors are generated, such as the husband assumes a higher proportion of housework, female employees receive a certain replenishment of time and energy resources in the family interface. When the husband takes up a high proportion of housework, the incremental resources given to the female employee may offset or even surplus the resources depleted by the female employee's desire to have children, which ultimately affects the relationship between the female employee's desire to have children and the wellbeing she experiences. Based on the premise that contexts (e.g., family support contexts) have a moderating effect (54), the following hypothesis is proposed as shown in Figure 1.

**Hypothesis 8**. The relationship between female employees' fertility intentions and female employees' wellbeing is positively moderated by the proportion of husbands taking up housework, i.e., the higher the proportion of husbands taking up housework, the stronger the effect of female employees' fertility intentions on their wellbeing.

## 4. Research methods

### 4.1. Object and characteristics of the study

This study was based on the classical work-family conflict scale and improved according to the requirements of the research elements. The reliability and validity of the scales met the expectations in the pre-small-scale pre-testing phase. In the large sample testing phase, the validity of the scale in this cross-sectional study was 412. They had different occupational backgrounds (Teachers, finance, lawyers, government employees, etc.) and came from eight provinces (Hunan, Guangdong, Fujian, Shanxi, etc.) in China. They were all working married female employees (age range 24–44 years) in dual-earner households in China. The participants were recruited through different WeChat (which is the most popular social software in China) communities which have been joined by researchers in previous social activities. When selecting the sample, the female interviewees were initially interviewed and collected information such as work status, marital status and fertility. Women who are not currently working, have no husband (whether widowed or divorced), and have no plans to have children are excluded from the final interview list. The researcher sent online questionnaires to those who agreed to participate in the study and asked the respondents to send the questionnaires to the other spouse to obtain the required research data. Data were collected for 1 month from early to late January 2022, and participants who completed the survey received an online cash envelope of 10 RMB as a reward.

Dual-earner couples each completed the questionnaire, and the data from both spouses were matched by marking each other's microsignals for data screening at a later stage. Husband-completed scales included personal information (used as a control variable for data processing at a later stage).

### 4.2. Measurement tools

This study involved data collection on the dimensions of work-family conflict, fertility intentions, work stress, and employee wellbeing, mainly by developing scale items based on established domestic and international scales. The Work-Family Conflict Scale and the Conflict Coping Strategies Scale used in this study were both English scales, which were translated by a graduate student in English and an associate professor in English, and the translation results were exchanged for back translation. The scale was scored on a 5-point Likert scale from 1 to 5, with a score of 1 for strongly disagreeing and 5 for strongly agreeing.

#### 4.2.1. Work-family conflict scale

Referring to the Work-Family Conflict Scale (WFCS) compiled by Carlson et al. (56), the scale consists of two main dimensions: work-family conflict (WFC) and family-work conflict (FWC), with a total of 18 items. The work-family conflict dimension in this study includes a three-factor model based on time, stress and behavior, including nine items such as “I have to miss family activities because I have to spend a lot of time at work,” and the Cronbach's alpha of this dimension is 0.975. The family-work conflict dimension includes a three-factor model based on time, stress, and behavior, including nine items such as “The amount of time I spend on family responsibilities often prevents me from fulfilling my work responsibilities,” with a Cronbach's alpha value of 0.959. The work-family conflict dimension was measured in this study. The Cronbach's alpha for the overall scale was 0.967; the KMO spherical test coefficient was 0.958, *p* < 0.05. The results of the validation factors for this scale were χ^2^/df = 2.473, CFI = 0.972, TLI = 0.958, RMSEA = 0.051, and SRMR = 0.026. In summary, the reliability indicators and model fit indicators of the overall scale and subscales of work-family conflict were as expected, indicating that the scale has good reliability and validity and is suitable for further research analysis.

#### 4.2.2. Fertility intention scale

Based on Miller (28), Bailey (57), and Zheng (29) quantitative studies on fertility intentions, a scale was developed to investigate female employees' fertility intentions in the context of the “comprehensive two-child” and “three-child” policies and the epidemic situation. The scale was developed to investigate the fertility intention of female employees in the context of the “comprehensive two-child” and “three-child” policies and the epidemic. The scale consists of three dimensions: the number of desired children, the gender of desired children, and the intensity of desire, with 14 questions. In this study, the number of desired children dimension includes “I think I need to have 2 or 3 children to make my life complete and my family happy.” The Cronbach's study also included three questions. The Cronbach's alpha value of this dimension is 0.948, the dimension of desire for children's gender contains 4 items such as “I want to have both children,” and its Cronbach's alpha value is 0.976, and the dimension of intensity of desire contains “The Cronbach's alpha value was 0.976”. The Cronbach's α value for the 7 question items such as “I will postpone my childbirth plan if the epidemic continues” was 0.965. The Cronbach's α value for the overall scale of female employees' desire to have children measured in this study was 0.963; the KMO spherical test coefficient was 0.985, *p* < 0.05. The test results for the validation factor of this scale were χ^2^/df = 3.216, CFI = 0.968, TLI = 0.948, RMSEA = 0.025, and SRMR = 0.041. In summary, the reliability indicators and model fit indicators of the overall scale and sub-scales of work-family conflict were as expected, indicating that the scale has good reliability and validity and is suitable for further research analysis.

#### 4.2.3. Wellbeing scale

The scale consists of five dimensions: psychological stress, intention to leave, job satisfaction, family satisfaction, and leisure satisfaction, with 22 questions. In this study, the psychological stress dimension includes “I always feel very stressed when there are uncertainties or problems to be solved in my work or life.” The Cronbach's alpha value for this dimension was 0.962. The intention to leave dimension included “I am now looking for other jobs or job opportunities offered by other companies.” The Cronbach's alpha for this dimension is 0.957; the job satisfaction dimension includes “I am satisfied with the current working atmosphere and colleagues' relationship.” The Cronbach's alpha value was 0.971 for 3 items, and the family satisfaction dimension included “I feel satisfied with the relationship between my family members.” The Cronbach's alpha value was 0.966 for the 4 questions, and the leisure satisfaction dimension included “I currently have quality leisure.” The Cronbach's alpha value for the overall work-family conflict scale was 0.965; the KMO spherical test coefficient was 0.952, *p* < 0.05. The results of the validation factor of the scale were χ^2^/df = 3.482, CFI = 0.979, TLI = 0.961, RMSEA = 0.049, and SRMR = 0.038. In summary, the reliability indicators and model fit indicators of the overall scale and sub-scales of work-family conflict were as expected, indicating that the scale has good reliability and validity and is suitable for further research analysis.

#### 4.2.4. Working pressure gauge

Based on the work stress scale compiled by Price and Spence (30), a flexible work stress scale was developed for conducting work stress research during the epidemic. The scale consists of four main dimensions: work load, role conflict, role ambiguity, and lack of resources, with a total of eight questions. In this study, the workload dimension includes “During the epidemic, my workload did not exceed my ability to cope.” The Cronbach's alpha value for this dimension was 0.955. The role conflict dimension included “During the epidemic, my work demands from different supervisors were often in conflict.” The Cronbach's alpha for this dimension was 0.979. The role ambiguity dimension included “During the epidemic, I often did not know what my job entailed and the steps I needed to take to complete it.” The Cronbach's α value for the two questions was 0.950; the under-resourced dimension included “During the epidemic, I had a high degree of time freedom to complete my work.” The Cronbach's α value for the overall work-family conflict scale measured in this study was 0.966; the KMO spherical test coefficient was 0.974, *p* < 0.05. The results of the validation factor for this scale were χ^2^/df = 2.594, CFI = 0.931, TLI = 0.970, RMSEA = 0.024, and SRMR = 0.036. In summary, the reliability indicators and model fit indicators of the overall scale and sub-scales of work-family conflict were as expected, indicating that the scale has good reliability and validity and is suitable for further research analysis.

Response options were scored on a five-point Likert scale ranging from “strongly disagree” 1 to “strongly agree” 5. High scores indicate that couples in dual-earner households are more stressed about childcare, and a few reverse items require shifting of the data to assess their scores. The age, education, job type, and monthly income of wives in dual-earner families were included as control variables in the analysis for data processing.

#### 4.2.5. Control variables

In this study, age, education level, type of work, and monthly income were put into the model as control variables to exclude their interference with the results.

### 4.3. Descriptive statistics

According to the statistics of this study, the average age of female employees interviewed is 34.48 years old, and 74% of the interviewees are aged 25–45 years old, which indicates that female employees are at a high age of work-family conflict. The education level is mostly “college” and “bachelor's degree,” accounting for 69.47% of the total number, indicating that the interviewed female employees generally have a higher education level. The difference in monthly income of female employees is obvious, with the main samples distributed in two classes: below RMB 4,000 and RMB 4,000–8,000, accounting for 68.13% of the total number of employees, and 7.42% of the total number of employees earning more than RMB 15,000. Regarding the work pressure of husbands during the epidemic, those who got 4 points and above after reversing the reverse question accounted for 20.83% of the respondents, which means that more husbands still suffered from greater work pressure during the epidemic. The proportion of housework undertaken by working husbands is relatively scattered, with the highest proportion being 26.48% who “undertake a medium (30%−50%) proportion of housework.” The number of years of working experience of female employees was mainly distributed in the range of “6 to 10 years” and “11 to 15 years,” accounting for 65.73% of the total number of employees.

### 4.4. Homologous deviation test

Although the questions of this scale were divided into two parts and answered by female employees and their husbands separately, which reduced the probability of homophily to a certain extent, it is not known whether the data were actually answered by female employees and their husbands separately because the data were obtained by filling out the questionnaire online during the epidemic period in this study, so homophily may still exist. To test for homoscedasticity bias, Harman's one-way analysis of variance was used. The results showed that the unrotated first principal component analysis factor explained 36.741% of the variance, which was <40%, i.e., indicating that there was no serious homozygosity bias in this study.

### 4.5. Analysis methods

Descriptive and correlation analyses were performed in this study using SPSS 24.0 software to examine the means, standard deviations, and correlations among the study variables and hypothesis testing. Path analysis was conducted using AMOS 20.0 software to assess the model fit, and this study used the comparative fit index (CFI; values >0.95 indicate adequate model fit) and root mean square error of approximation (RMSEA; values <0.06 indicate adequate model fit). In addition, to improve the reliability of the mediating effect findings, Mplus 7.4 was used to estimate confidence intervals (CIs) to test the mediating effects assumed in this study using Bootstrap method with bias correction. The mediating effect was significant at the 0.05 level if the 95% CI did not contain zero (58). To account for missing values (0–2% of each variable), this study used maximum likelihood estimates as default values in the path analysis (59).

## 5. Empirical analysis

### 5.1. Discriminant validity test

The validated factor analysis (CFA) was used to test the discriminant validity. As shown in Table 1, the fitted data of the six-factor model (χ^2^/df = 1.863, CFI = 0.991, TLI = 0.987, RMSEA = 0.045, SRMR = 0.021) were significantly better than the other competing models, thus indicating that the six variables had better discriminant validity among them and could be analyzed in the next step of the test.

**Table 1:** Results of validation factor analysis.

**Results of validation factor analysis (Models)**	**χ^2^/df**	**CFI**	**TLI**	**RMSEA**	**SRMR**
Six factors (WFC, FWC, WEB, INT, PRE, HOU)	1.863	0.991	0.987	0.038	0.025
Five factor (WFC + FWC, WEB, INT, PRE, HOU)	3.256	0.903	0.878	0.046	0.077
Five factors (PRE + HOU, WFC, FWC, WEB, INT)	3.197	0.924	0.914	0.054	0.079
Four factors (WFC + FWC + INT, WEB, PRE, HOU)	4.265	0.917	0.906	0.062	0.071
Four factors (WFC + FWC + WEB, INT, PRE, HOU)	4.378	0.902	0.895	0.069	0.076
Three factors (WFC + FWC + INT, PRE + HOU, WEB)	5.261	0.851	0.827	0.073	0.085
Three factors (WFC + FWC + WEB, PRE + HOU, INT)	6.342	0.836	0.819	0.077	0.088
Two-factor (WFC + FWC + WEB + INT, PRE + HOU)	7.115	0.788	0.765	0.081	0.083
Two-factor (WFC + FWC + WEB + INT + HOU, PRE)	8.647	0.673	0.651	0.085	0.094
Single factor (WFC + FWC + WEB + INT + PRE + HOU)	9.336	0.665	0.649	0.104	0.112

WEB, women employees' wellbeing; INT, women employees' willingness to have children; PRE, husband's flexible work pressure; HOU, husband's share of housework; WFC, women employees' work-family conflict; FWC, women employees' family-work conflict.

### 5.2. Descriptive statistics

The correlation coefficients of the six variables were analyzed by SPSS 24.0 and the correlation coefficients between the variables are shown in Table 2. According to the Table 2, age was significantly and positively correlated with monthly income (R = 0.134, *p* < 0.01); education level was significantly and positively correlated with monthly income (R = 0.241, *p* < 0.001); flexible work pressure during husband's epidemic was significantly and negatively correlated with husband's share of housework (R = −0.491, *p* < 0.01); husband's share of housework was significantly positively correlated with fertility intention (R = 0.046, *p* < 0.001), significantly negatively correlated with female employees' work → family conflict (R = −0.113, *p* < 0.01), significantly negatively correlated with female employees' family → work conflict (R = −0.134, *p* < 0.01), and significantly negatively correlated with female employees' wellbeing (R = 0.228, *p* < 0.01) was significantly negatively related to female employees' wellbeing (R = 0.228, *p* < 0.01). Fertility intention was significantly negatively related to female employees' work-family conflict (R = −0.142, *p* < 0.01), significantly negatively related to female employees' family-work conflict (R = −0.103, *p* < 0.01), and significantly positively related to female employees' wellbeing (R = 0.255, *p* < 0.01). Work-family conflict of female employees is significantly and positively related to family-work conflict of female employees (R = 0.457, *p* < 0.001) and significantly and negatively related to wellbeing of female employees (R = −0.233, *p* < 0.001). Female employees' family → work conflict was significantly and negatively related to female employees' wellbeing (R = −0.421, *p* < 0.001). The above results are consistent with expectations and lay a good foundation for the follow-up study.

**Table 2:** Correlation of variables.

**Variables**	**M**	**SD**	**1**	**2**	**3**	**4**	**5**	**6**	**7**	**8**	**9**	**10**
1. Age	34.481	7.258	–									
2. Education level	2.339	0.658	0.081	–								
3. Working years	8.155	8.426	0.272	0.175	–							
4. Monthly income	4,239.167	8.164	0.134^**^	0.241^***^	0.185^**^	–						
5. Flexible work pressure during the husband's epidemic	2.683	0.912	−0.015	−0.023	−0.082	0.023	–					
6. Husband's share of housework	3.124	0.815	−0.016	0.096	−0.055	0.042	−0.491^***^	–				
7. Female employees' willingness to give birth	2.631	1.031	−0.198	−0.027	0.076	0.034	−0.092	0.046^***^	–			
8. Female employees work → family conflict	2.825	1.208	−0.082	0.052	0.028	0.015	0.123	−0.113^**^	−0.142^**^	–		
9. Female employees' family → work conflict	2.967	1.156	−0.027	0.018	0.063	−0.036	0.014	−0.134^**^	−0.103^**^	0.457^***^	–	
10. Wellbeing of female employees	3.082	1.112	0.063	0.074	0.021	0.017	−0.136	0.228^**^	0.255^**^	−0.233^***^	−0.421^***^	–

^**^p < 0.01; ^***^p < 0.001.

### 5.3. Hypothesis testing

#### 5.3.1. Mediating effect test

This study first used SPSS to conduct cascade regression of variables to validate the relationship between work → family conflict for female employees, family → work conflict for female employees, fertility intentions of female employees, and wellbeing of female employees, with fertility intentions of female employees as the mediating variable. After that, the robustness of the mediating effect of the model was again verified using the suggestion of Hayes (60), using Mplus7.4 with 5,000 put-back sampling through Bootstrap.

In the first step, we take female employees' fertility intention as the dependent variable and put control variables in model 1 to exclude the interference of control variables on female employees' fertility intention and other variables; in the second step (i.e., model 2), we put female employees' work-family conflict in model 1 to examine the influence of female employees' work-family conflict on female employees' fertility intention; in the third step (i.e., model 3), we put female employees' family-work conflict in model 1 to examine the influence of female employees' family-work conflict on female employees' fertility intention. Step 4 (i.e., model 4) puts female employees' wellbeing as the dependent variable and puts control variables in the model to exclude their interference with female employees' wellbeing and other variables; Step 5 (i.e., model 5) puts female employees' work-family conflict on the basis of model 4 to examine the effect of female employees' work-family conflict on fertility intention. conflict on female employees' wellbeing; the sixth step (i.e., model 6) puts female employees into family → work conflict on the basis of model 4 to examine the impact of female employees' family → work conflict on female employees' wellbeing; the seventh step (i.e., model 7) puts female employees' fertility intention variable on the basis of model 4 to examine the direct impact of female employees' fertility intention on female employees' wellbeing; the eighth step (i.e., model 8). Put the variable of female employees' work → family conflict on the basis of model 7 to examine the mediating effect of female employees' work → family conflict on female employees' wellbeing through fertility intention; Step 9 (i.e., model 9): put the variable of female employees' family → work conflict on the basis of model 7 to examine the mediating effect of female employees' family → work conflict on female employees' wellbeing through fertility intention (as shown in Table 3—the results of the mediating effect test are shown in the graph).

**Table 3:** Results of the test for mediating effects of fertility intention.

	**Category**	**Fertility intention**				**Wellbeing**			**Wellbeing**	
		**Model 1**	**Model 2**	**Model 3**	**Model 4**	**Model 5**	**Model 6**	**Model 7**	**Model 8**	**Model 9**
Independent variable	Work → Family conflict		−0.317^**^			−0.283^**^			−0.185^**^	
	Family → Work conflict			−0.288^**^			−0.279^**^			−0.196^**^
Intermediate variables	Fertility intention							0.328^*^	0.276^**^	0.261^**^
Control variables	Age	0.065	0.054	0.071	0.128	0.147	0.138	0.136	0.141	0.139
	Level of education	−0.039	−0.043	−0.047	0.243	0.184	0.176	0.173	0.166	0.171
	Working years	0.032	0.038	0.035	0.047	0.053	0.043	0.044	0.051	0.048
	Monthly income	0.121	0.134	0.129	0.133^**^	0.128^**^	0.137^**^	0.131^**^	0.129^**^	0.125^**^
*R* ^2^		0.031^*^	0.142^**^	0.118^**^	0.049^*^	0.175^**^	0.158^**^	0.168^**^	0.188^**^	0.179^**^
Δ*R*^2^			0.111^**^	0.087^**^		0.126^**^	0.109^**^	0.119^**^	0.020^**^	0.011^**^

The above work → family conflict, family → work conflict, childbirth intention, age, education level, job type, and monthly income are all data of female employees.

^*^p < 0.05; ^**^p < 0.01.

The results of model 2 show that the regression coefficient of female employees' work → family conflict on fertility intention is −0.317, *p* < 0.01, which indicates that there is a significant negative relationship between female employees' work → family conflict and fertility intention, and H1a is verified. This proves the reality that female employees feel more work-to-family conflict in the workplace and have lower fertility intentions, which is in line with the current social reality in China. For example, work takes up a lot of time and there is no time to get pregnant; work is too intense and there is no intention to get pregnant; workplace competition is fierce and female employees want to hold on to their hard-earned workplace status, while pregnancy will cause female employees to withdraw from the workplace for a short time to the point of losing their workplace advantage.

The results of model 3 show that the regression coefficient of female employees' family → work conflict on female employees' fertility intention is −0.288, *p* < 0.01, indicating that there is a significant negative correlation between female employees' family → work conflict and female employees' fertility intention, and H1b is verified. However, the absolute value of this correlation coefficient is smaller than H1a, which indicates that the impact of family life conflict on work on female employees' fertility intention is smaller compared to the impact on female employees' fertility intention due to work-to-family conflict. For example, female employees have to spend a lot of time and energy to take care of the elderly at home after work, and this situation will make female employees exhausted or tired in work, which will affect her willingness to get pregnant and have children, because pregnancy and children will intensify the above conflicts and make female employees feel stressful in work and life.

Model 5 shows that after controlling for the effect of relevant demographic variables on female employees' wellbeing, the regression coefficient of work → family conflict on female employees' wellbeing is −0.283, *p* < 0.01, indicating that the effect of work → family conflict on female employees' wellbeing is negatively and significantly correlated, and H3a is verified. This hypothesis is also consistent with the actual situation. When female employees often work overtime so much that they have no time to enjoy spending time with their children and husbands, and when female employees are under too much work pressure and have negative emotions overflowing to bad-mouth family members to the extent that it affects family relationships, all of these situations will affect female employees' feelings and experiences of wellbeing.

Model 6 shows that the regression coefficient of family-work conflict on female employees' wellbeing is −0.279, *p* < 0.01, indicating a negative and significant relationship between family-work conflict and female employees' wellbeing, which is verified by H3b. This hypothesis is confirmed by the reality that when female employees need to spend a lot of time and energy to take care of young children at home so that they cannot accept and challenge the promotion opportunities given by their leaders at work, this will affect the perception of wellbeing of female employees.

Model 7 shows that when controlling for the effect of demographic variables on female employees' wellbeing, the regression coefficient of fertility intention on female employees' wellbeing is 0.328, *p* < 0.05, i.e., fertility intention significantly and positively affects female employees' wellbeing, which is verified by H2. This may be because, based on Chinese social ethics, most women subconsciously perceive that having children is an important aspect to prove their family value or social value. In Chinese society, a woman who is married and delays having children will be under pressure from her neighbors and friends; the difference between male and female births can create a huge disparity in women's family status in some areas. Therefore, most female employees have the idea that having a strong fertility and a healthy and smart baby can prove their value and gain recognition and respect from their families and society. Even the idea of “even if you have three children, you must have a boy” is deeply rooted in the minds of many female employees, especially those from rural areas.

Model 8 shows that after adding female employees' work → family conflict to model 7, the effect of female employees' fertility intention on female employees' wellbeing is still significant, with a regression coefficient of 0.276, *p* < 0.01. However, the regression coefficient of female employees' work → family conflict on female employees' wellbeing changes from −0.283, *p* < 0.01 in model 5 to −0.185, *p* < 0.01, with lower absolute value. Based on the above analysis, it can be seen that fertility intention plays a partially mediating role in the relationship between female employees' work → family conflict and female employees' wellbeing, and H4a was verified.

Model 9 shows that after adding female employees' family → work conflict to model 7, the effect of female employees' fertility intention on female employees' wellbeing is still significant, with a regression coefficient of 0.261, *p* < 0.01. However, the regression coefficient of female employees' family → work conflict on female employees' wellbeing changes from −0.279, *p* < 0.01 in model 6 to −0.196, *p* < 0.01, with lower absolute value. Based on the above analysis, it can be seen that fertility intention plays a partially mediating role in the relationship between female employees' family → work conflict and female employees' wellbeing, and H4b is verified.

Next, as suggested by Hayes (60), in order to further confirm the mediating effect, this study continued to use Bootstrap multiple sampling method with 5,000 put-back samples of the sample data. If the 95% confidence interval does not contain 0, the mediation effect is again proven to exist. The results showed that the indirect effect value of female employees' work → family conflict through fertility intention on female employees' wellbeing was 0.121 with a standard error of 0.032 and a 95% confidence interval of (0.040, 0.182), which did not contain 0. H4a was again verified. The indirect effect value of female employees' family → family conflict on female employees' wellbeing through fertility intention is 0.116, standard error is 0.035, 95% confidence interval is (0.051, 0.213), does not contain 0, and H4b is verified again.

#### 5.3.2. Moderating effect test

By placing the control and independent variables into the model in steps, when controlling for the effect of demographic variables on the proportion of husbands who undertake housework, we obtain the results in Table 4. From the results, we can see that the regression coefficient of husband's flexible work pressure on the husband's share of housework is −0.335, *p* < 0.01. It shows that husband's flexible work pressure has a significant negative correlation on the husband's share of housework, and H5 is verified.

**Table 4:** Test results of direct effect of husband's elastic stress and the husband's share of housework.

	**Category**	**Husband's share of housework**	
		**Model 1**	**Model 2**
Independent variable	Husband's flexible work pressure		−0.335^**^
Control variables	Age (husband)	0.121	0.173
	Level of education (husband)	0.329^**^	0.277^**^
	Working years (husband)	0.058	0.046
	Monthly income (husband)	−0.147	−0.138
*R* ^2^		0.079^**^	0.263^**^
Δ*R*^2^			0.184^**^

** *p* < 0.01.

In Table 5, Model 12 examines the moderating effect of husband's share of housework on the relationship between work-family conflict and fertility intention. Model 14 examines the moderating effect of husband's share of housework on the relationship between family-work conflict and fertility intention. Model 17 test the moderating effect of husband's share of housework on the relationship between fertility intention and female employees' wellbeing.

**Table 5:** Test results of direct effect of husband's elastic stress and the husband's share of housework.

	**Category**	**Fertility intention**					**Female employee wellbeing**		
		**Model 10**	**Model 11**	**Model 12**	**Model 13**	**Model 14**	**Model 15**	**Model 16**	**Model 17**
Main effect	Work → Family conflict		−0.305^**^	−0.248^**^					
	Family → Work conflict				−0.276^**^	−0.313^**^			
	Fertility intention							0.344^*^	0.689^**^
	Husband's share of housework		0.068	0.087	0.070	0.195		0.218	0.204
Moderating effects	Work → family conflict × husband's share of housework			−0.105^**^					
	Family → Work conflict × husband's share of housework					−0.117^**^			
	Husband's share of housework × fertility intention								0.265^**^
Control variables	Age	0.065	0.052	0.041	0.162	0.153	0.128	0.134	0.143
	Level of education	−0.039	−0.042	−0.04	−0.044	−0.041	0.243	0.172	0.169
	Working years	0.032	0.035	0.033	0.031	0.025	0.047	0.041	0.055
	Monthly income	0.121	0.13	0.122	0.126	0.111	0.133^**^	0.199^**^	0.134^**^
*R* ^2^		0.031^*^	0.147^**^	0.199^**^	0.124^**^	0.149^**^	0.049^*^	0.170^**^	0.186^**^
Δ*R*^2^			0.116^**^	0.052^**^	0.093^**^	0.025^**^		0.121^**^	0.016^**^

^*^p < 0.05; ^**^p < 0.01.

The results of model 12 showed that the interaction term of work → family conflict and husband's share of housework had a significant negative effect on female employees' fertility intentions, β = −0.105, *p* < 0.01; the results of model 14 showed that the interaction term of family → work conflict and husband's share of housework had a significant negative effect on female employees' fertility intentions, β = −0.117, *p* < 0.01; model 17 results showed that the interaction term between the husband's share of housework and Fertility intention had a significant positive effect on female employees' wellbeing, β = 0.265, *p* < 0.01.

**Figure 2:**
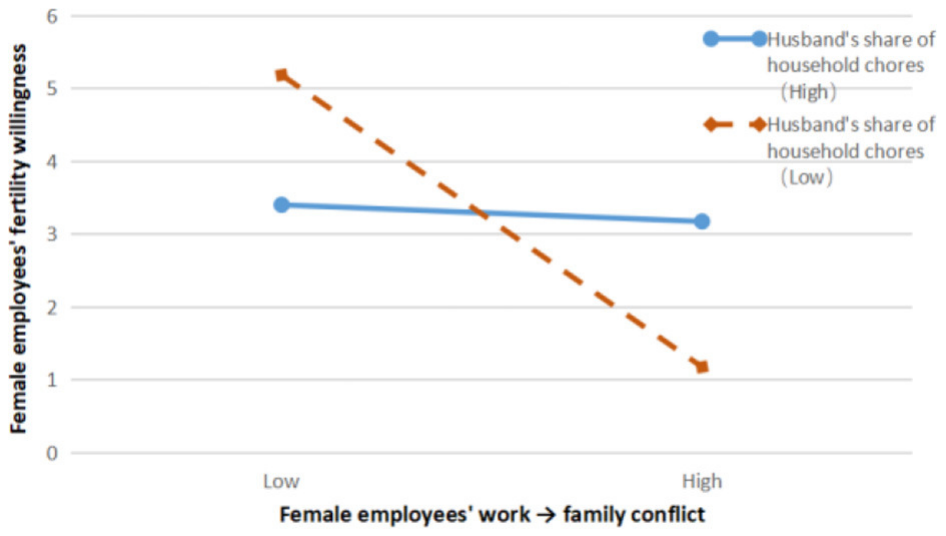
Moderating effect of husband's share of housework on the relationship between work → family conflict and fertility intention of female employees.

Next, the moderating effect of husband's share of housework was plotted based on the suggestion of Aiken and West (61) to classify the level of husband's share of housework as the mean plus or minus one standard deviation. The moderating effect of husband's share of housework on the relationship between work-family conflict and female employees' fertility intentions is shown in Figure 2, and the moderating effect of husband's share of housework on the relationship between family-work conflict and female employees' fertility intentions is shown in Figure 2. The negative effect of female employees' work → family conflict on fertility intention is not significant when the proportion of husbands taking up housework is high, β = −0.069, *p* = n.s.; when the proportion of husbands taking up housework is low, the negative effect of female employees' work → family conflict on fertility intention is stronger, β = −0.473, *p* < 0.001, and H6a is verified. This is consistent with the social experience, when the husband takes on a higher proportion of housework, female employees even if they experience higher work → family conflict, for example, mothers are unable to fulfill their duties as parents to take their children to and from school because of the long commute to and from work and the long work commute, but because the husband takes on a higher proportion of housework, picking up and dropping off children can be done by the husband when the wife is unable to do so, which makes mothers who feel guilty about their first-born children will still have a higher desire to have another child.

**Figure 3:**
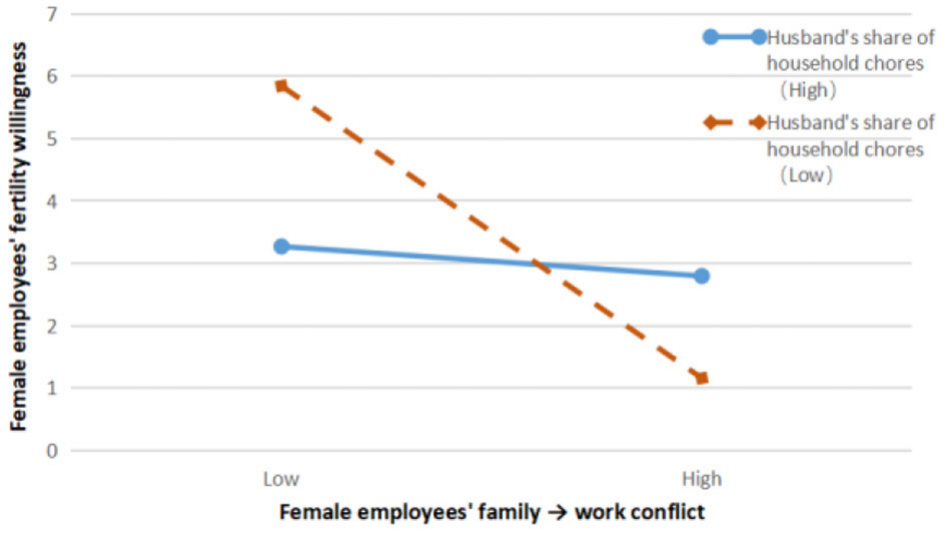
Moderating effect of husband's share of housework on the relationship between family → work conflict and fertility intention of female employees.

In Figure 3, the negative effect of female employees' family → work conflict on female employees' fertility intentions is not significant when the proportion of husbands taking up housework is high, β = −0.071, *p* > 0.1; when the proportion of husbands taking up housework is low, the negative effect of female employees' family → work conflict on female employees' fertility intentions is strong, β = −0.486, *p* < 0.001, H6b was verified. This validation is also consistent with reality. For example, female employees suffer from workplace marginalization and even salary adjustment because their children are weak and sick at home and they often have to take time off during working hours to take their children to the doctor, while their husbands have a lower proportion of family responsibilities and obligations because they are always away on business trips, so that female employees have a stronger intention not to have children again.

**Figure 4:**
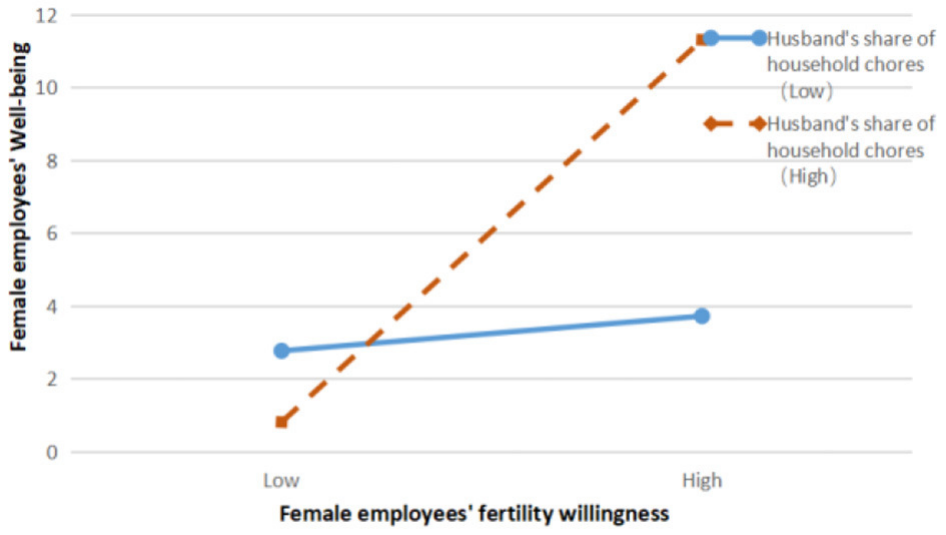
Moderating effect of husband's share of housework on the relationship between female employees' fertility intention and wellbeing.

In Figure 4, the positive effect of fertility intention on female employees' wellbeing is significant when the proportion of husbands taking up housework is high, β = 0.492, *p* < 0.001; when the proportion of husbands taking up housework is low, the positive effect of fertility intention on female employees' wellbeing is not significant, β = 0.113, *p* > 0.1, and H8 is verified. Reflecting to the reality, in Chinese society, it is generally believed that marriage must be for the purpose of childbearing, and couples should have at least one child as the family fertility goal. Therefore, there is a certain primitive willingness motivation for female employees to have children, whether due to social pressure or pressure from elders within the family. The strong willingness to have children gives hope to the family, so much so that many couples improve their relationship during pregnancy preparation, couples move together toward one family goal—having children, and female employees' wellbeing in the family field is enhanced. At the same time, due to the spillover effect, the good mood of female employees at home with their husbands will also be brought to the workplace, which will to a certain extent optimize the workplace work status and workplace socialization of female employees, and their wellbeing in the workplace will be enhanced to a certain extent. When the husband takes a high proportion of family responsibilities at home, it generally means that the husband has a strong sense of family responsibility and family belonging. This will make the female employee think that having children is not her business alone, and the stronger her willingness to have children, the stronger the wellbeing she feels in a situation where her husband takes on a higher proportion of household duties.

In order to test the mediating effect of the two stages of husband's share of housework being moderated, this study used Bootstrap method for 5,000 put-back sampling of the sample data to estimate the confidence intervals for the indirect effect values under high and low husband's share of housework and the difference between the indirect effect values under high and low husband's share of housework. This is shown in Table 6. As shown in Table 6, the indirect effect value of female employees' work → family conflict affecting female employees' wellbeing through fertility intention is −0.003 with a standard error of 0.026 and a 95% confidence interval of [−0.080, 0.036], including 0, when the husband's share of housework is low; when the husband's share of housework is high, the indirect effect value of female employees' work → family conflict affecting female employees' wellbeing through fertility intention The indirect effect value of female employees' work → family conflict through fertility intention is 0.171, standard error is 0.058, 95% confidence interval is [0.042, 0.277], which does not contain 0. The difference between the indirect effect at high and low levels is −0.174, standard error is 0.064, 95% confidence interval is [−0.298, −0.033], which does not contain 0, indicating that the indirect effect at high and low levels. The difference is significant. The above results suggest that the proportion of husbands taking up housework positively moderates the indirect effect of female employees' work → family conflict on female employees' wellbeing through fertility intention, and H7a is verified.

**Table 6:** Results of the test for mediating effects of being moderated at two stages (work-family conflict for female employees as independent variables).

**Regulated variables husband's share of housework**	**Female employees' work-family conflict (X)**→**Fertility intention (M)**→**Women employees' wellbeing (Y)**			
	**Indirect effects**	**Standard error**	**95% confidence interval**	
			**Lower limit**	**Upper limit**
Low husband's share of housework	−0.003	0.026	−10.080	0.035
High husband's share of housework	0.171	0.058	0.042	0.277
Difference	−0.174	0.064	−0.298	−0.033

As shown in Table 7, the indirect effect of family-work conflict affecting female employees' wellbeing through fertility intention is −0.008 with standard error 0.031 and 95% confidence interval [−0.076, 0.029], including 0, when the proportion of husbands taking up housework is low; the indirect effect of family-work conflict affecting female employees' wellbeing through fertility intention is 0.169 with standard error 0.055 and 95% confidence interval [0.038, 0.280], when the proportion of husbands taking up housework is high. The difference between the indirect effects at the high and low levels is −0.177, with a standard error of 0.068 and a 95% confidence interval of [−0.302, −0.041], which does not contain 0. The difference between the indirect effects at the high and low levels is −0.177, with a standard error of 0.068 and a 95% confidence interval of [−0.302, −0.041], which does not contain 0. The difference is significant. The above results suggest that the proportion of husbands taking up housework positively moderates the indirect effect of family → work conflict on female employees' wellbeing through female employees' fertility intentions, as verified by H7b.

**Table 7:** Results of the test for mediating effects of being moderated at two stages (family-work conflict for female employees as independent variables).

**Regulated variables husband's share of housework**	**Female employees' family-work conflict (X)**→**Fertility intention (M)**→**Women employees' wellbeing (Y)**			
	**Indirect effects**	**Standard error**	**95% confidence interval**	
			**Lower limit**	**Upper limit**
Low husband's share of housework	−0.008	0.031	−0.076	0.029
High husband's share of housework	0.169	0.055	0.038	0.280
Difference	−0.177	0.068	−0.302	−0.041

## 6. Conclusion

Based on the conservation of resource theory, this study constructs a two-stage mediated model of female employees' work-family conflict affecting female employees' wellbeing being regulated. The results show that work-family conflict (work-family conflict and family-work conflict) negatively affects fertility intention; fertility intention positively affects female employees' wellbeing; fertility intention mediates the relationship between female employees' work-family conflict and female employees' wellbeing; the proportion of husbands taking up housework moderates the relationship between female employees' work-family conflict and fertility intention The relationship between fertility intention and female employees' wellbeing is positively moderated, which in turn moderates the mediating role of fertility intention in the relationship between female employees' work-family conflict and female employees' wellbeing. According to the validation results, all hypotheses were supported by the data.

The results of this study on the impact of work family conflict on childbearing intention are similar to those of Minello et al. (62) on the impact of work family conflict on childbearing of academic teacher mothers. The conclusion of this article on the impact of fertility on female employees' wellbeing confirms Liu and Zhou (63)'s view in the research on the positive relationship between Chinese mothers' second child birth and their wellbeing. The conclusion on the direct effect of work family conflict on happiness in this paper is consistent with that of Carnevale and Hatak (64). Based on the unique evolution of fertility policy in China's national conditions and the public crisis background of the COVID-19 epidemic in a special period, this study takes Chinese female employees as samples, selects variables from the family level, considers the intermediary effect of fertility desire on the relationship between work family conflict and happiness, and takes the proportion of husband's share of household chores as a moderator variable (at the same time, considers the impact of the epidemic background on husband's work pressure under flexible working conditions). This innovative research is a supplement and improvement to the previous research on the relationship between work family and happiness.

### 6.1. Theoretical contributions

First, this study broadens the research perspective of the work-family interface. In recent years, in order to gain a more comprehensive understanding of the impact of work-family relationships on employees, an important trend in work-family conflict research from a work perspective is to examine the negative effects of work resources (e.g., social support, schedule control, etc.) and work demands (e.g., work overload, job insecurity, role conflict, time pressure, etc.) on employees in the workplace based on the conservation of resource theory (65–70). However, work-family conflict does not only originate from the work interface, but also from the family interface (71). Therefore, exploring work-family relationships should consider not only work elements but also family elements (72). In this study, we shift the perspective of the study to explore the influencing factors from the family perspective and find that an important indicator of the family element, fertility intention, is affected by work-family conflict and thus affects female employees' wellbeing. This finding contributes to a more comprehensive understanding of the influencing mechanism of work-family conflict, and also enlightens researchers on work-family relationships to examine the role of employee characteristics or behaviors from both work and family perspectives in a dialectical and comprehensive manner.

Second, it enriches the elements of work-family interface research. Previous studies examined the negative effects of work-family conflict on individual wellbeing of one spouse alone (73), ignoring the possible positive effects of their interaction. The conservation of resource theory suggests that individuals will adopt defensive resource strategies to reduce their resource investment in other domains when facing resource loss (74). Both spouses provide resources to the family in a complementary manner, and when the wife needs to consider whether “childbearing” in the family will lead to a loss of family resources, the husband immediately gives more resources—providing more time and energy for housework—as a supplement to family resources, the wife does not feel the loss of family resources at this time and does not adopt a defensive resource strategy, i.e., she does not reduce her resources in other areas outside the family—“work” resource investment.

Third, the impact mechanisms and mitigation conditions of work-family conflict in the context of the COVID-19 new crown epidemic were explored, expanding the application of the conservation of resource theory. Previous studies have less considered the impact of the epidemic on work-family conflict (65). In particular, the impact of the epidemic on work family conflict is seldom considered from the perspective of family factors. This study found that the impact of the epidemic on the husband's work status will further affect the female employee's (wife's) work family conflict, in other words, the flexible work mechanism under the epidemic exposes husbands to a different kind of work stress than before—flexible work stress. According to the conservation of resource theory, individuals always act out of a code of conduct to protect their existing resources from further losses (75). The flexible work stress experienced by husbands causes husbands to feel the depletion of their time and energy resources and affects their time and energy investment in family matters. The results of this study suggest that the interaction between husband and wife attenuates the negative effects of each and thus enhances the overall effectiveness of the family, providing empirical support for the application of the conservation of resource theory to the work-family interface in the context of the epidemic. Furthermore, all data of this study were collected during the epidemic, this makes all sample characteristics have epidemic attribute, so this paper expands the work family research during the epidemic period to a certain extent.

### 6.2. Practical insights

First, managers should consider not only the direct effects of policies when formulating policies, but also the indirect effects that policies may have on other family members of employees. Most studies have focused on the direct effects of corporate policies on employees' work status and work-family conflict, but based on the conservation of resource theory, the employment policies that male employees encounter in the company will affect their resource allocation in the family domain and thus their family performance. As the family is a business place where both spouses share the same goals, both spouses need to work together to ensure that no loss of total family resources occurs, so the treatment male employees receive in the firm will affect their wives' contribution to the family and further affect their wives' workplace performance. There is ample evidence that a positive work-family relationship not only fits with the organization's social responsibility to focus on employee wellbeing, but also has a positive impact on employee performance in the organization.

Second, managers should develop management policies during an epidemic that are more responsive to the actual needs of employees during an epidemic. In the post-epidemic era, the epidemic will probably coexist with humans for a long time. Unlike the management patterns in other countries, China has adopted dynamic clearance measures to track the epidemic. A typical practice of this management measure is to take the form of restricting the movement of people to block the virus transmission channels and control the spread of the virus. Public health management policies have posed new challenges to business management. Many companies adopted a flexible work system during the epidemic, where employees completed their work targets at home and their performance was assessed on the corporate line. Flexible work stress, an exogenous influence indicator in this study, has been verified in this study for its impact on employees' work at home during the epidemic. Therefore, this paper suggests that under the normalization of the epidemic, it is necessary for enterprises to adopt diverse supervision mechanisms, work patterns, and assessment methods to urge employees to complete their established performance during the implementation of flexible work office, such as online live office, online live meetings, and online approval of daily work content, in order to improve employees' work efficiency and motivation, so as to ensure employees' work performance while optimally easing employees' relationship with family members.

Third, the management of female employees should give due consideration to the family status of female employees. For example, if female employees of marriage and childbearing age have demands for childbirth, they can be recruited to balance the ratio of female employees and stagger the maternity leave of female employees by age staggering to avoid the shortage of labor caused by the concentration of female employees in childbirth. When enterprises can flexibly deal with the problems of female employees' work absence due to their family and social responsibilities, female employees of enterprises will be respected and treated favorably by enterprises and put more enthusiasm into their work, and enterprises will get more benefits and output from female employees, and finally realize the win-win situation of improving employees' wellbeing and increasing enterprises' performance.

Fourth, enterprises should recognize the importance of childcare for female employees. Most employed women in childbearing age (25–39 years old) may withdraw from the labor market due to marriage and raising children (76). At the same time, enterprises can improve the employment competitiveness of female employees and attract high-quality female employees for re-employment through training and re-employment, so as to make up for their skills decline when they leave the labor market. In this regard, the maternity leave policy promoted by China in recent years, that is, 158 days of maternity leave is available for all children who give birth to one or two children while on duty, and 15 days of maternity leave is added for normal and difficult childbirth (cesarean section or twins). At the same time, wages and salaries during the leave are paid by maternity insurance. However, business executives may not support this policy (especially in terms of the length of leave for female employees) due to the shortage of staff. Therefore, human resource managers can consider holding regular symposiums or meetings with supervisors of different business departments in the enterprise to coordinate employees' work family conflicts, so as to help supervisors gain insight into employees' work and life trends, solve employees' work family conflicts, and ultimately have a positive effect on the organization's retention of valuable employees who have been recruited and trained. In addition, improve the organization's employee care policy, for example, to accommodate employees who occasionally have to leave school to pick up their children or take their elderly parents to the hospital in advance, which may also reduce the work family conflict of female employees.

### 6.3. Research limitations and future research directions

Although this paper has some theoretical and practical merits, there are still some limitations that can continue to be improved in subsequent studies, and there are some valuable questions and research directions that can continue to be advanced in subsequent studies.

First, homology bias was controlled by optimizing the procedure of data collection. The data used in this study were cross-sectional data, and considering the epidemic factor, this study collected data through the Internet, and there were certain industry and regional differences in the samples obtained, while this study collected research scales from female employees and their husbands separately by means of marker matching. However, due to a certain time lag in the relationship between the antecedent variables and the mediating, moderating and outcome variables, the use of data collection between two time points is more likely to avoid Homology bias. Also adopting a follow-up study can further test how the dynamic process of relationship change between couples affects one partner's work-family conflict to further deepen the understanding of the conservation of resource theory at the work-family conflict interface. In addition, future studies can use various means of data acquisition, such as experimental methods, to enhance the external validity and internal validity of the results.

Second, other strategic relationships that exist between work-family conflict and wellbeing can be explored. This study only explored female employees' fertility intentions as a factor influencing female employees' family roles, but there are many factors in the family domain that may influence work-family conflict, such as the health status of family members, widowed or divorced family status, etc. Conducting research and examining different family factors can help to understand the influence mechanism of work-family conflict more comprehensively. In addition, research on how male employees' family role behaviors are influenced by female employees' work factors is also a direction for future researchers to study in depth.

Third, the positive factors of work-family conflict on work are not explored enough. Previous studies have focused on the negative effects of work-family conflict on employees' work, but neglected to explore its positive effects. Chinese people are traditionally known as a hard-working and kind working people. After experiencing work-family conflict, Chinese employees find that they cannot achieve excellence in work or take care of their families by investing their time and energy in work and family in a balanced way. From this perspective, the more intense the work-family conflict is, the more likely it is that employees will devote more enthusiasm and energy to their work, helping the company to achieve growth and efficiency. Fully examining the positive consequences of work-family conflict in either the work domain or the family domain has practical implications for enriching theory and guiding practice, and warrants more in-depth research by future researchers.

## Data availability statement

The original contributions presented in the study are included in the article/supplementary material, further inquiries can be directed to the corresponding author.

## Author contributions

ZZ and JM contributed to conception and design of the study. DL organized the database. YS performed the statistical analysis. ZZ wrote the first draft of the manuscript. JM and YM wrote sections of the manuscript. All authors contributed to manuscript revision, read, and approved the submitted version.

## References

[1] Epifanio MS Andrei F Mancini G Agostini F Piombo MA Spicuzza V. The impact of COVID-19 pandemic and lockdown measures on quality of life among Italian general population. J Clin Med. (2021) 10:289. 10.3390/jcm10020289 33466778 PMC7830623

[2] Grabowski J Stepien J Waszak P Michalski T Meloni R Grabkowska M. Social isolation during COVID-19 pandemic perceived stress and containment measures compliance among polish and italian residents. Front Psychol. (2021) 12:673514. 10.3389/fpsyg.2021.673514 34122269 PMC8194265

[3] Ying T Wang K Liu X Wen J Goh E. Rethinking game consumption in tourism: a case of the 2019 novel coronavirus pneumonia outbreak in China. Tourism Recreat Res. (2021) 46:304–9. 10.1080/02508281.2020.1743048

[4] Lins S Aquino S Costa AR Koch R. From panic to revenge: compensatory buying behaviors during the pandemic. Int J Social Psychiatry. (2022) 68:921–2. 10.1177/00207640211002557 33719662

[5] Gap GG. Report 2020. Geneva: World Economic Forum (2020).

[6] Xie X. The impact of two-child policy on China's fertility level-based on logit model. Saudi J Econ Fin. (2021) 5:100–6. 10.36348/sjef.2021.v05i03.001

[7] Greenhaus JH Beutell NJ. Sources of conflict between work and family roles. Acad Manage Rev. (1985) 10:76–88. 10.5465/amr.1985.4277352

[8] Carlson D Kacmar KM Zivnuska S Ferguson M Whitten D. Work-family enrichment and job performance: a constructive replication of affective events theory. J Occup Health Psychol. (2011) 16:297. 10.1037/a0022880 21728437

[9] Begall K Mills M. The impact of subjective work control, job strain and work–family conflict on fertility intentions: a European comparison. Eur J Popul. (2011) 27:433–56. 10.1007/s10680-011-9244-z 22131613 PMC3208813

[10] Cerrato J Cifre E. Gender inequality in housework and work-family conflict. Front Psychol. (2018) 9:1330. 10.3389/fpsyg.2018.01330 30123153 PMC6086200

[11] Mikolajczak M Raes ME Avalosse H Roskam I. Exhausted parents: sociodemographic, child-related, parent-related, parenting and family-functioning correlates of parental burnout. J Child Fam Stud. (2018) 27:602–14. 10.1007/s10826-017-0892-4

[12] Huizink AC Menting B De Moor MHM Verhage ML Kunseler FC Schuengel C. From prenatal anxiety to parenting stress: a longitudinal study. Arch Women's Mental Health. (2017) 20:663–72. 10.1007/s00737-017-0746-5 28634716 PMC5599437

[13] Moreira H Fonseca A Caiado B Canavarro MC. Work-family conflict and mindful parenting: the mediating role of parental psychopathology symptoms and parenting stress in a sample of Portuguese employed parents. Front Psychol. (2019) 10:635. 10.3389/fpsyg.2019.00635 30967822 PMC6438855

[14] Abidin RR. The determinants of parenting behavior. J Clin Child Psychol. (1992) 21:407–12. 10.1207/s15374424jccp2104_12

[15] Rindfuss RR Brewster KL Kavee AL. Women, work, and children: behavioral and attitudinal change in the United States. Popul Dev Rev. (1996) 457–82. 10.2307/213771628392967

[16] Kim JS Bang H. Education fever: Korean parents' aspirations for their children's schooling and future career. Pedagogy Cult Soc. (2017) 25:207–24. 10.1080/14681366.2016.1252419

[17] Seek Lee C Ui Park S Kyoung Hwang Y. The structural relationship between mother's parenting stress and child's well-being: the mediating effects of mother's growth mindset and hope. Indian J Sci Technol. (2016). 9:102702. 10.17485/ijst/2016/v9i36/102702

[18] Van Bakel HJ Van Engen ML Peters P. Validity of the parental burnout inventory among Dutch employees. Front Psychol. (2018) 9:697. 10.3389/fpsyg.2018.00697 29875711 PMC5974116

[19] Hwang W Kim S. Husbands' childcare time and wives' second-birth intentions among dual-income couples: The mediating effects of work–family conflict and parenting stress. J Soc Serv Res. (2021) 47:850–9. 10.1080/01488376.2021.1936746

[20] Huang C. An analysis of the impacts of universal two-child policy on urban women's career development in China: taking 420 professional women in Shenyang city as the sample. Open J Soc Sci. (2018) 6:343–53. 10.4236/jss.2018.611026

[21] Yoon SY Lim S Kim L. Labour market uncertainty and the economic foundations of marriage in South Korea. Asian Popul Stud. (2022) 18:6–23. 10.1080/17441730.2021.1932065

[22] Liu J Liu T. Two-child policy, gender income and fertility choice in China. Int Rev Econ Fin. (2020) 69:1071–81. 10.1016/j.iref.2018.12.009

[23] Schnettler B Orellana L Miranda-Zapata E Saracostti M Poblete H Lobos G. Diet quality during the COVID-19 pandemic: effects of workplace support for families and work-to-family enrichment in dual-earner parents with adolescent children. Appetite. (2022) 169:105823. 10.1016/j.appet.2021.105823 34822922 PMC8611499

[24] Bontrager M Clinton MS Tyner L. Flexible work arrangements: a human resource development tool to reduce turnover. Adv Dev Human Resour. (2021) 23:124–41. 10.1177/1523422320982930

[25] Rapisarda S Ghersetti L Girardi D De Carlo NA Corso LD. Smart working and online psychological support during the covid-19 pandemic: Work-family balance, well-being, and performance. InPACT. (2021) 301–6. 10.36315/2021inpact062

[26] Schmid L Wörn J Hank K Sawatzki B Walper S. Changes in employment and relationship satisfaction in times of the COVID-19 pandemic: evidence from the German family Panel. Eur Soc. (2021) 23(sup1):S743–58. 10.1080/14616696.2020.1836385

[27] Schieman S Badawy PJ Milkie M Bierman A. Work-life conflict during the COVID-19 pandemic. Socius. (2021). 7:2378023120982856. 10.1177/2378023120982856

[28] Miller WB. Differences between fertility desires and intentions: implications for theory, research and policy. In: Vienna Yearbook of Population Research. (2011), *p*. 75–98. 10.1553/populationyearbook2011s7532672529

[29] Zheng S. A Study on the Influence of Second Child Fertility Intention Based on Literature Measurement and Questionnaire Survey (Master's thesis). Guizhou University of Finance and Economics (2021). 10.27731/d.cnki.ggzcj.2021.000015

[30] Price L Spence SH. Burnout symptoms amongst drug and alcohol service employees: gender differences in the interaction between work and home stressors. Anxiety Stress Coping. (1994) 7:67–84. 10.1080/10615809408248394

[31] Hobfoll SE. Conservation of resources: a new attempt at conceptualizing stress. Am Psychol. (1989) 44:513. 10.1037/0003-066X.44.3.513 2648906

[32] Ma W Jin J. Study on the impact of work-family conflict on the second child fertility intention of nurses of childbearing age. Health Res. (2021) 3, 268–272. 10.19890/j.cnki.issn1674-6449.2021.03.007

[33] Wu F. Research on fertility intention: theory and demonstration. Sociol Res. (2020) 35:218–240+246. 10.19934/j.cnki.shxyj.2020.04.011

[34] Diener E Emmons RA. The independence of positive and negative affect. J Pers Soc Psychol. (1984) 47:1105. 10.1037/0022-3514.47.5.1105 6520704

[35] Boehm JK Lyubomirsky S. Does well-being promote career success? J Career Assess. (2008) 16:101–116. 10.1177/1069072707308140

[36] Hofmann W Luhmann M Fisher RR Vohs KD Baumeister RF. Yes, but are they happy? Effects of trait self-control on affective well-being and life satisfaction. J Personal. (2014) 82:265–77. 10.1111/jopy.12050 23750741

[37] Hosie P Willemyns M Sevastos P. The impact of well-being on managers' contextual and task performance. Asia Pac J Human Resour. (2012) 50:268–87. 10.1111/j.1744-7941.2012.00029.x

[38] Liu C Peng Y Rubenstein K. Challenge job demands, time-based work–family conflict, and family well-being outcomes: The moderating effect of conscientiousness. Int J Stress Manag. (2022) 29:182. 10.1037/str0000242

[39] Castro Torres AF Pesando LM Kohler HP Furstenberg F. Family change and variation through the lens of family configurations in low-and middle-income countries. Popul Space Place. (2022) 28:e2531. 10.1002/psp.2531 PMC11192499 38912222

[40] Nie J. Are the rural elderly happier with more children—Also on the impact of intergenerational support on the subjective well-being of rural elderly. J Northwest Univ. (2018) 48:91–101. 10.16152/j.cnki.xdxbsk.2018-06-010

[41] Qiu Y. Research on Reproductive Behavior and Subjective Well-Being of People of Childbearing Age (Master's thesis). University of Electronic Science and Technology (2021). 10.27005/d.cnki.gdzku.2021.002321

[42] Ugur ZB. Does having children bring life satisfaction in Europe? J Well-being Stud. (2020) 21:1385–406. 10.1007/s10902-019-00135-5

[43] Mu Z Xie Y. The impact of childbirth on parents' subjective well-being. Sociol Res. (2014) 6:124–47. 10.19934/j.cnki.shxyj.2014.06.006

[44] Leng C Chen Q. Research on the relationship between the number of children and the well-being of the elderly—an empirical analysis based on cgss2013. J Dalian Univ Technol. (2019). 40:60–8. 10.19525/j.issn1008-407x.2019.05.008

[45] Michel JS Hargis MB. Linking mechanisms of work–family conflict and segmentation. J Vocat Behav. (2008) 73:509–22. 10.1016/j.jvb.2008.09.005

[46] Michel JS Kotrba LM Mitchelson JK Clark MA Baltes BB. Antecedents of work–family conflict: a meta-analytic review. J Organ Behav. (2011) 32:689–725. 10.1002/job.695

[47] Li F Zheng L Xu L. Parental support in the context of the three child policy: a comparison of mechanisms inside and outside the family. Learn Pract. (2022) 131−40. 10.19624/j.cnki.cn42-1005/c.2022.02.012

[48] Li Y. Research on improving the well-being of grassroots postal employees with humanistic care. Postal Res. (2020) 53–4. 10.13955/j.cnki.yzyj.2020.03.028

[49] Zhang H. Research on the Relationship Between Work-Family Conflict, Subjective Well-Being and Job Performance of Employees in Z Company. (Master's thesis). Anhui University of Finance and *Economics*. (2020). 10.26916/d.cnki.gahcc.2020.000644

[50] Loi R Xu AJ Chow CW Kwok JM. Customer misbehavior and store managers' work-to-family enrichment: the moderated mediation effect of work meaningfulness and organizational affective commitment. Hum Resour Manage. (2018) 57:1039–48. 10.1002/hrm.21883

[51] Wu F Li J. Three signals that China is facing the risk of fertility crisis: low fertility rate, low fertility intention and fertility deficit. J Shanxi Normal Univ. (2022) 61–8. 10.16207/j.cnki.1001-5957.20211211.004

[52] Shifrin NV Michel JS. Flexible work arrangements and employee health: a meta-analytic review. Work Stress. (2022) 36:60–85. 10.1080/02678373.2021.1936287

[53] Yue L. Flexible working system: demand, problems and countermeasures. Res Trade Union Theory. (2022) 41–53. 10.19434/j.cnki.ghllyj.2022.01.00412320982

[54] Meyer RD Dalal RS Hermida R. A review and synthesis of situational strength in the organizational sciences. J Manage. (2010) 36:121–40. 10.1177/0149206309349309

[55] Talukder AMH. Supervisor support and organizational commitment: the role of work–family conflict, job satisfaction, and work–life balance. J Employ Counsel. (2019) 56:98–116. 10.1002/joec.12125

[56] Carlson DS Kacmar KM Williams LJ. Construction and initial validation of a multidimensional measure of work–family conflict. J Vocat Behav. (2000) 56:249–76. 10.1006/jvbe.1999.1713

[57] Bailey MJ. Reexamining the impact of family planning programs on US fertility: Evidence from the war on poverty and the early years of Title X. American Economic Journal: Applied Economics. (2012) 4.2:62–97. 10.1257/app.4.2.62 22582135 PMC3348617

[58] Preacher KJ James PS. Advantages of Monte Carlo confidence intervals for indirect effects. Communication Methods and Measures. (2012) 6.2:77–98. 10.1080/19312458.2012.679848

[59] Acock AC. Working with missing values. J Marriage Fam Couns. (2005) 67.4:1012–28. 10.1111/j.1741-3737.2005.00191.x

[60] Hayes A. Introduction to mediation, moderation, and conditional process analysis. J Educ Measure. (2013) 51:335–7. 10.1111/jedm.1205035923144

[61] Aiken LS West SG. Multiple Regression: Testing And Interpreting Interactions. London: Sage (1991). 10.1037/0021-9010.84.6.897

[62] Minello A Martucci S Manzo LK. The pandemic and the academic mothers: present hardships and future perspectives. Eur Soc. (2021) 23(sup1):S82–94. 10.1080/14616696.2020.1809690

[63] Liu J Zhou Z. Mothers' subjective well-being after having a second child in current China: a case study of Xi'an city. Int J Environ Res Public Health. (2019) 16:3823. 10.3390/ijerph16203823 31658744 PMC6843609

[64] Carnevale JB Hatak I. Employee adjustment and well-being in the era of COVID-19: Implications for human resource management. J Bus Res. (2020) 116:183–7. 10.1016/j.jbusres.2020.05.037 32501303 PMC7241356

[65] Molina JA. The work–family conflict: Evidence from the recent decade and lines of future research. J Fam Econ Issues. (2021) 42:4–10. 10.1007/s10834-020-09700-0

[66] Dinh H Martin A Leach L Strazdins L Nicholson J Allen T. Is self-employment a good option? Gender, parents and the work-family interface. Sex Roles. (2021) 84:731–46. 10.1007/s11199-020-01195-1

[67] Hong X Liu Q Zhang M. Dual stressors and female pre-school teachers' job satisfaction during the COVID-19: the mediation of work-family conflict. Front Psychol. (2021) 12:691498. 10.3389/fpsyg.2021.691498 34168602 PMC8217622

[68] Hu S Jiang L Probst TM Liu M. The relationship between qualitative job insecurity and subjective well-being in Chinese employees: the role of work–family conflict and work centrality. Econ Ind Democr. (2021) 42:203–25. 10.1177/0143831X18759793

[69] Haar J Brougham D. Work antecedents and consequences of work-life balance: A two sample study within New Zealand. Int J Human Resour Manage. (2022) 33:784–807. 10.1080/09585192.2020.1751238

[70] Hjálmsdóttir A Bjarnadóttir VS. “I have turned into a foreman here at home”: families and work–life balance in times of COVID-19 in a gender equality paradise. Gender Work Organ. (2021) 28:268–83. 10.1111/gwao.12552 33041540 PMC7537149

[71] Fisher J Languilaire JC Lawthom R Nieuwenhuis R Petts RJ Runswick-Cole K. Community, work, and family in times of COVID-19. Commun Work Fam. (2020) 23:247–52. 10.1080/13668803.2020.1756568

[72] Uddin M. Addressing work-life balance challenges of working women during COVID-19 in Bangladesh. Int Soc Sci J. (2021) 71:7–20. 10.1111/issj.12267 34230685 PMC8251227

[73] Maulida AF Widiaty I. Work family conflict on female teachers in the time of the COVID-19 Pandemic. In: 4th International Conference on Innovation in Engineering and Vocational Education (ICIEVE 2021). Atlantis Press (2022). p. 176–9. 10.2991/assehr.k.220305.037

[74] Darouei M Pluut H. Work from home today for a better tomorrow! How working from home influences work-family conflict and employees' start of the next workday. Stress Health. (2021) 37:986–99. 10.1002/smi.3053 33887802 PMC9291295

[75] Mao Y He J Morrison AM Andres Coca-Stefaniak J. Effects of tourism CSR on employee psychological capital in the COVID-19 crisis: from the perspective of conservation of resources theory. Curr Issues Tourism. (2021) 24:2716–34. 10.1080/13683500.2020.1770706

[76] Akgunduz YE Janneke P. Child care prices and maternal employment: A metaanalysis. J Econ Surv. (2018) 32.1:118–33. 10.1111/joes.12192

